# Pharmacologic Inhibition of MLK3 Kinase Activity Blocks the *In Vitro* Migratory Capacity of Breast Cancer Cells but Has No Effect on Breast Cancer Brain Metastasis in a Mouse Xenograft Model

**DOI:** 10.1371/journal.pone.0108487

**Published:** 2014-09-29

**Authors:** Kun Hyoe Rhoo, Megan Granger, Joynita Sur, Changyong Feng, Harris A. Gelbard, Stephen Dewhurst, Oksana Polesskaya

**Affiliations:** 1 Department of Microbiology and Immunology, University of Rochester Medical Center, Rochester, New York, United States of America; 2 Center for Neural Development and Disease, and Departments of Pediatrics and Neurology, University of Rochester Medical Center, Rochester, New York, United States of America; 3 Department of Biostatistics and Computational Biology, University of Rochester Medical Center, Rochester, New York, United States of America; Emory University, United States of America

## Abstract

Brain metastasis of breast cancer is an important clinical problem, with few therapeutic options and a poor prognosis. Recent data have implicated mixed lineage kinase 3 (MLK3) in controlling the *in vitro* migratory capacity of breast cancer cells, as well as the metastasis of MDA-MB-231 breast cancer cells from the mammary fat pad to distant lymph nodes in a mouse xenograft model. We therefore set out to test whether MLK3 plays a role in brain metastasis of breast cancer cells. To address this question, we used a novel, brain penetrant, MLK3 inhibitor, URMC099. URMC099 efficiently inhibited the migration of breast cancer cells in an *in vitro* cell monolayer wounding assay, and an *in vitro* transwell migration assay, but had no effect on *in vitro* cell growth. We also tested the effect of URMC099 on tumor formation in a mouse xenograft model of breast cancer brain metastasis. This analysis showed that URMC099 had no effect on the either the frequency or size of breast cancer brain metastases. We conclude that pharmacologic inhibition of MLK3 by URMC099 can reduce the *in vitro* migratory capacity of breast cancer cells, but that it has no effect on either the frequency or size of breast cancer brain metastases, in a mouse xenograft model.

## Introduction

Brain metastases occur in 10–16% of patients with breast cancer [1], and present limited therapeutic options [2] with a mean one-year survival rate of only about 20% [3]. Therefore, it is necessary to develop new therapies to treat metastatic disease in clinical setting.

Brain metastasis a multi-step process that involves entry of cells from the primary tumor site into the blood stream (intravasation), trapping of cells in cerebral blood vessels and, finally, exit from blood vessels into brain parenchyma (extravasation) and tumor formation from subsequent cell proliferation [4]. The process of tumor cell extravasation is thought to be rate-limiting for metastasis [4], [5], and involves cell migration across the endothelial barrier and through the basement membrane underneath [5], [6]. Thus, cell migratory capacity is believed to be an important predictor of metastatic potential [5]. As a result, *in vitro* cell wounding assays [7] and transwell migration assays have been used to measure the migratory activity of tumor cells, and to help infer one important aspect of their metastatic potential [8].

Cell migration results from multiple intracellular events, resulting in cytoskeletal rearrangement and changes in focal adhesion systems [9], [10]. Recently, the upstream mitogen activated kinase kinase kinase (MAP3K), mixed lineage kinase 3 (MLK3) has been implicated in the regulation of cell migration [11], [12], [13], [14], and MLK3 has been shown to be highly expressed in human breast cancer cell lines [8]. Moreover, MLK3 knockdown or pharmacologic inhibition of MLK3 reduced the migratory activity of breast cancer cells in *in vitro* wound healing and transwell migration assays [8], [15]. MLK3 knockdown in MDA-MB-231 cells also prevented *in vivo* metastasis of these cells from the breast fat pad to the lung [15] and to distant lymph nodes, by inhibiting both cell growth and cell migration [11]. However, pharmacologic inhibitors of MLK3 kinase activity have not been previously evaluated in experimental animal models for breast cancer metastasis. This is an important omission because shRNA-mediated gene knockdown affects all functions of MLK3 (including both kinase and scaffolding activities), whereas pharmacologic inhibition of MLK3 selectively affects only the kinase activity of the protein. We therefore used a novel, brain penetrant, MLK3 inhibitor URMC099 [16], [17] Our results show that URMC099 efficiently inhibited the migration of breast cancer cells in an *in vitro* cell wounding assay and in *in vitro* transwell migration assay, but that it had no effect on *in vitro* cell growth. We also assessed the effect of URMC099 on tumor formation in a mouse xenograft model of breast cancer brain metastasis [18]. Our data revealed that URMC099 had no effect on either the frequency or size of breast cancer brain metastases. We conclude that pharmacologic inhibition of MLK3 reduces the *in vitro* migratory capacity of breast cancer cells, but that it has no effect on either the frequency or size of breast cancer brain metastases, when tested in a preclinical mouse xenograft model.

## Materials and Methods

### Cell Cultures

MDA-MB-231, MCF10A, and HS578t cells were obtained from the American Type Culture Collection. MDA-MB-231 EGFP8.4 cells were obtained from Dr. Patricia S. Steeg at the National Cancer Institute. The construction of this cell line is described in [18]. Briefly, a brain homing clone of MDA-MD-231 cells [19] was stably transfected with a plasmid expressing the enhanced green fluorescent protein (eGFP), yielding line MDA-MB-231-BR-EGFP8.4; this cell line is referred to in the text as EGFP8.4. MDA-MB-231, MCF10A, HS578t, and EGFP8.4 cells were maintained in Dulbecco’s Modified Eagle’s Medium (DMEM) supplemented with 10% of fetal bovine serum (FBS), 100 units of penicillin, 100 µg of streptomycin and 292 µg/mL of L-glutamine (PSG).

### Wound Healing Assay

To test the inhibitory effect of URMC099 on cell migration *in vitro*, a scratch wound healing assay was performed [8]. Four cell lines were used: MDA-MB-231, a metastatic human epithelial breast adenocarcinoma cell line that is tumor-forming in immunosuppressed mice [20]; eGFP8.4, a subline of MDA-MB-231 that is “brain homing” and stably transfected with eGFP; HS578t, a human breast carcinoma cell line that is also tumorigenic in immunosuppressed mice [21]; and MCF10A, a normal mammary epithelial cell line that is not tumor-forming (although it does form colonies in semi-solid media [22]). Cells were plated at a density 5.0×10^5^ cells/mL in media specified above, and allowed to grow until confluence. Then, a scratch wound was introduced in each well using a 200 µL pipet tip, and media was replaced with media containing mitomycin-C (1 µg/mL) plus either URMC099 or vehicle. Images of the wound were taken at 48 hours post scratch; for each image, the width of the wound was measured at five places equally distributed along the wound by the researcher blinded to the condition, and then the average width was calculated. Each well was imaged at three different parts of the scratch wound. The wound width in a given well was calculated as an average from these three measurements. For each well the wound width at the specified time was compared with the width immediately after scratch introduction (t = 0), and the wound healing recovery was calculated as follows: (1−(width of a wound at t/width of a wound at t = 0)*100). Then, the relative change of wound recovery was calculated by assigning 100% recovery to the condition that gave the highest level of wound recovery and 0% recovery to the condition that gave the lowest level of wound recovery.

### Transwell Migration Assay

The *in vitro* migration assay was conducted using a Boyden chamber transwell assay (8 µm pore size; Corning Costar, cat. #3422) with a polycarbonate membrane. Cells were starved in serum-free media overnight, then introduced into the upper chamber (5×10^4^ cell/ml) for 6 or 24 hours. For the 24-h assay, mitomycin-C was added to block cell proliferation. The lower chamber was filled with growth media supplemented with 10% FBS. After incubation the cells were fixed and stained by Diff-Quik (Siemens). Migrated cells in three random fields were counted; all conditions were performed in triplicate.

### Mouse Xenograft Model of Breast Cancer Brain Metastasis

The *in vivo* studies were approved by the IACUC of the University of Rochester, and conducted in compliance with local, state and federal regulations. The breast cancer brain metastasis model was performed as described [18]. Briefly, 6 to 8 week old female nu/nu mice were anesthetized by intraperitoneal injection of 100 mg/kg ketamine HCl and 10 mg/kg xylazine, and then inoculated with 100,000 cells in 0.1 mL of cold phosphate buffered saline (PBS) into the left ventricle. The next day, mice were injected intraperitoneally with URMC099 at a dose of 10 mg/kg, dissolved in 5% DMSO/45% saline/50% PEG400 to 2 mg/mL, or vehicle, twice daily for 20 days. On day 21 mice were sacrificed by CO_2_ suffocation. Brains were removed and fixed with 4% formaldehyde in PBS overnight, then transferred to 30% sucrose in PBS. The brains were then quickly frozen by immersing into isopentane cooled on dry ice. The frozen brains were sectioned coronally every 30 micrometers. Eight sections starting at bregma 2.0 and separated by 360 µm were mounted on glass slides for tumor evaluation under the microscope. The number of brain metastasis (BM) was counted by examining eGFP signals under a fluorescence microscope at 20X magnification. To determine the sizes of BM, pictures of 8–12 coronal sections per mouse were taken at 10x magnification, an the area of eGFP-positive BM was then measured using ImageJ software and Auto threshold algorithm (a version of IsoData algorithm [23]).

### Growth Rate of Breast Cancer Cells

Cells were seeded in a 24 well plate at an initial density of 5.0×10^4^ cells/mL in 0.5 ml of media. The cells were treated with either 200 µM of URMC099 or vehicle (0.002% DMSO). Cell number in each well was measured by trypsinizing the cells and counting them with a hematocytometer. The viability was tested by trypan blue dye exclusion. Each condition was tested in triplicate.

### Statistical Analyses

Mean values were compared using unpaired two-tailed t-test, using GraphPad Prism software. Permutation test was used to compare the growth rate of the cells.

## Results

### Pharmacologic Inhibition of MLK3 by URMC099 Reduces the Migratory Ability of Breast Cancer Cells, as Determined by an *In Vitro* Wound Healing Assay

MLK3 knockdown or pharmacologic inhibition of MLK3 has been shown to block the migratory activity of breast cancer cells in *in vitro* wound healing assays [8], [15], and to also prevent the *in vivo* metastasis of MDA-MB-231 cells from the breast fat pad to the lung [15] and to distant lymph nodes [11]. In light of these data, we first tested whether pharmacologic inhibition of MLK3 with a novel, brain penetrant, MLK3 inhibitor, URMC099 [16], [17], blocks the *in vitro* migratory activity of human breast cancer cells. To do this, we performed a scratch wound healing assay using four cell lines: MDA-MB-231, an invasive human breast cancer cell line; eGFP8.4, a subline of MDA-MB-231 that is stably transfected with GFP and “brain homing” in mice; HS578T, a tumorigenic human breast cancer cell line; and MCF10A, a non-transformed (though immortalized) human breast epithelial cell line.

The three cell lines chosen for analysis (MDA-MB-231, eGFP8.4 and Hs578t) are all triple-negative breast cancer cells. As such, they provide a model for TNBC - which can be aggressive and hard to treat, in part due to a lack of targeted treatments. We hypothesized that URMC099 might demonstrate activity against these tumor cells, by preventing their migration – and conducted experiments to test this hypothesis. We also tested the effect of URMC099 on migration of non-cancerous MCF10A cells.

**Figure 1:**
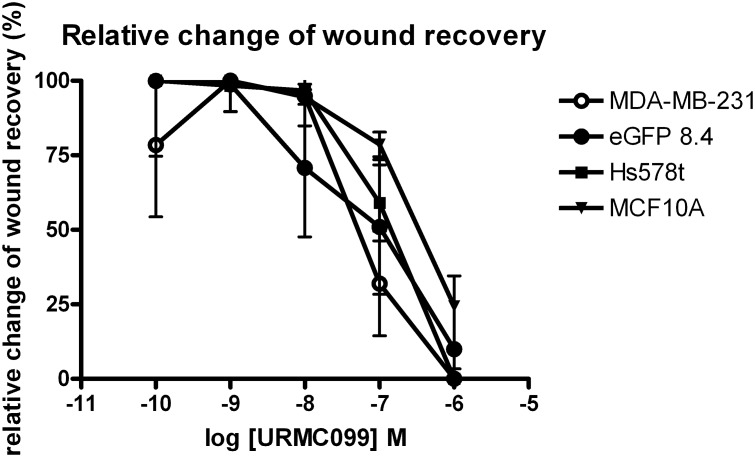
MLK3 inhibition decreases wound healing recovery in a dose dependent manner. Relative change of wound recovery was measured for MDA MB-231, eGFP8.4, HS578T and MCF10A cells. Each value is the mean of 4 wells, error bars denote the standard error of the mean; see Methods for details.

The relative change of *in vitro* wound recovery for all of these cell lines was decreased in the presence of URMC099, in a dose-dependent manner (Figure 1). In agreement with a report from the Gallo group [15], pharmacologic inhibition of MLK3 prevented the migration of MDA-MB-231 cells, and their “brain homing” subline, eGFP8.4 as well as Hs578t cells and non-tumorigenic human epithelial cells MCF10A (Figure 1). The IC_50_ values at 48 hrs are 123 nM (95% CI 37 nM to 411 nM) for MDA-MB-231, 95 nM (95% CI 4.58 nM to 1.37 µM) for eGFP8.4, 185 nM (95% CI 65 nM to 526 nM) for HS578t, and 231 nM (95% CI 126 nM to 424 nM) for MCF10A.

### The Migratory Ability of the Triple Negative Human Breast Cancer Cell Line, MDA-MB-231, and Its “Brain Homing” Subline eGFP8.4, Is Reduced by URMC099

**Figure 2:**
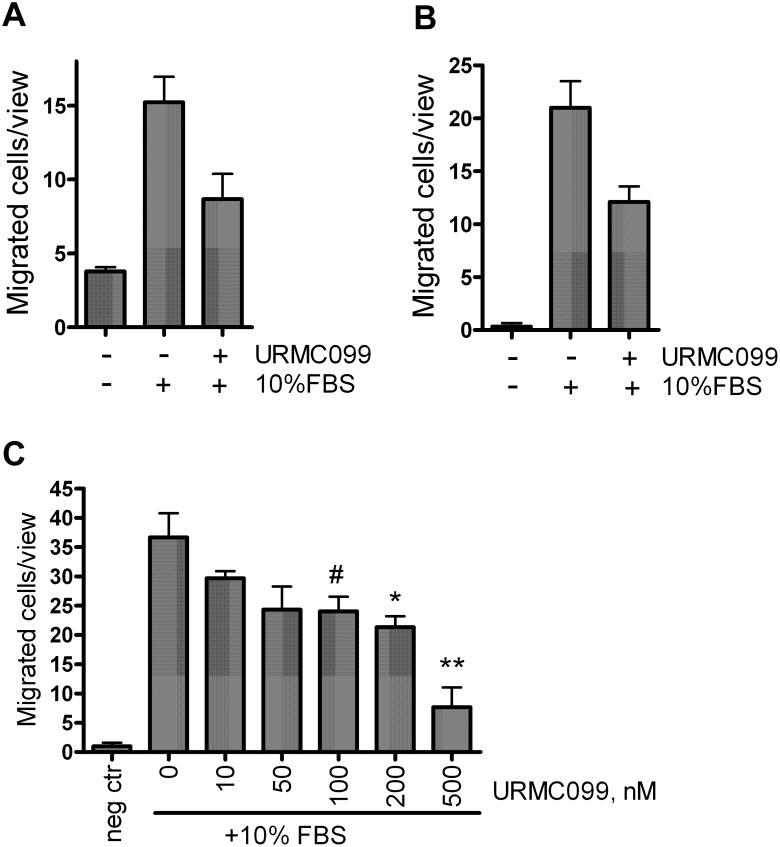
MLK3 inhibition decreases transwell migration of breast cancer cells. (**A**) MDA MB-231 or (**B**) eGFP8.4 migrated toward 10% FBS in presence of 200 nM URMC099 or vehicle during 24 hours. (**C**) eGFP8.4 cells migration toward 10% FBS reduced in dose-dependent manner; migration allowed for 6 hours. Each value is the mean of 3 wells, error bars denote the standard error of the mean. *, ** and # denote p<0.05 and p<0.005 and p = 0.058, two-tailed unpaired *t*-test.

As an additional way to test the inhibitory effect of URMC099 on cancer cell migration, an *in vitro* transwell migration assay was performed on MDA-MB-231 cells and eGFP8.4 cells (the latter being used in our subsequent *in vivo* experiments). In a 24-hour assay, we observed an approximately two-fold decrease in the number of migrated cells in the presence of URMC099, for both the MDA-MB-231 (Figure 2A), and eGFP8.4 (Figure 2B) cell lines. We also demonstrated that migration was reduced by URMC099 in a dose dependent manner, in the eGFP8.4 cell line (Figure 2C).

### Pharmacologic Inhibition of MLK3 by URMC099 Has no Effect on the *In Vitro* Growth of Breast Cancer Cells

**Figure 3:**
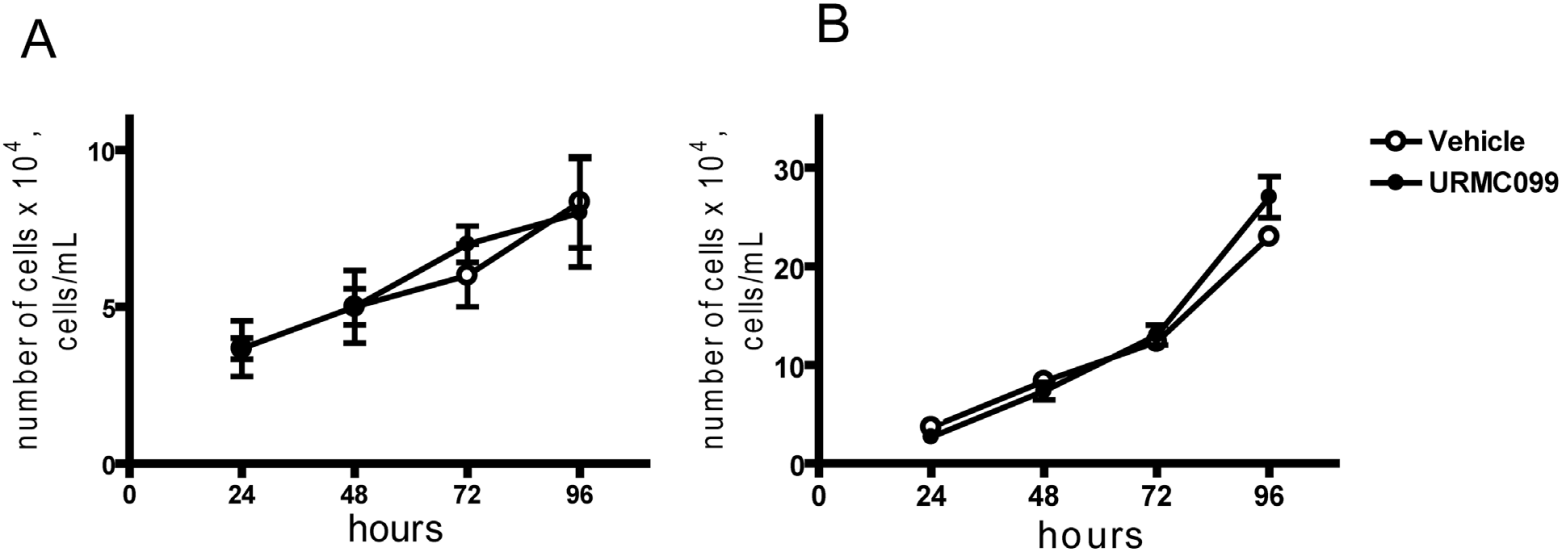
Growth rate of cancer cells *in vitro* is not affected by URMC099. (**A**) MDA MB-231 and (**B**) eGFP8.4 cells were cultured with either 200 nM URMC099 or vehicle. The cells were seeded at 5×10^4^ cells/mL initially in a 24 well plate and grown until they were confluent. Each data point represents the mean of quadruplicate wells, error bars denote standard deviation.

We next tested the effect of URMC099 on the *in vitro* growth of the “brain homing” MDA-MB-231 BR cells [19] expressing eGFP (eGFP8.4) and their parental cell line, MDA-MB-231. The cells were treated with either 200 nM URMC099 (a concentration equivalent to that in mice exposed to the drug) or vehicle alone. Cells treated with URMC099 grew at a similar rate to those treated with vehicle (Figure 3). Cell viability was >99% in all cases.

### Pharmacologic Inhibition of MLK3 Has no Effect on Breast Cancer Brain Metastasis Formation in a Mouse Xenograft Model

The effect of URMC099 on tumor formation *in vivo* was analyzed using a well characterized mouse xenograft model of breast cancer brain metastasis [18]. For these experiments, eGFP8.4 cells were inoculated into the left ventricle of immunodeficient *nu/nu* mice; animals were then treated with either URMC099 (10 mg/kg) or vehicle alone, every 12 hours for 20 days. This dose of URMC099 was chosen because it has been shown to be sufficient to effectively inhibit MLK3 in mice, with good penetration of the blood-brain barrier and potent inhibition of the phosphorylation of Jun N-terminal kinase (JNK) in brain tissue [16], [17]. On day 21 the mice were sacrificed and number of BM was assessed. Fifteen mice were used for each treatment group. BM were detected in 60% of mice, which is consistent with previous studies using this xenograft model by other investigators (Yuriy Shapovalov, personal communication). Thus, exposure to URMC099 did not reduce the proportion of mice that developed brain metastases. During the three weeks of treatment with URMC099 or vehicle no mortality or neurologic symptoms were observed. The mice treated with URMC099 gained weight similarly to the mice treated with vehicle, suggesting that the drug was well tolerated (data not shown).

**Figure 4:**
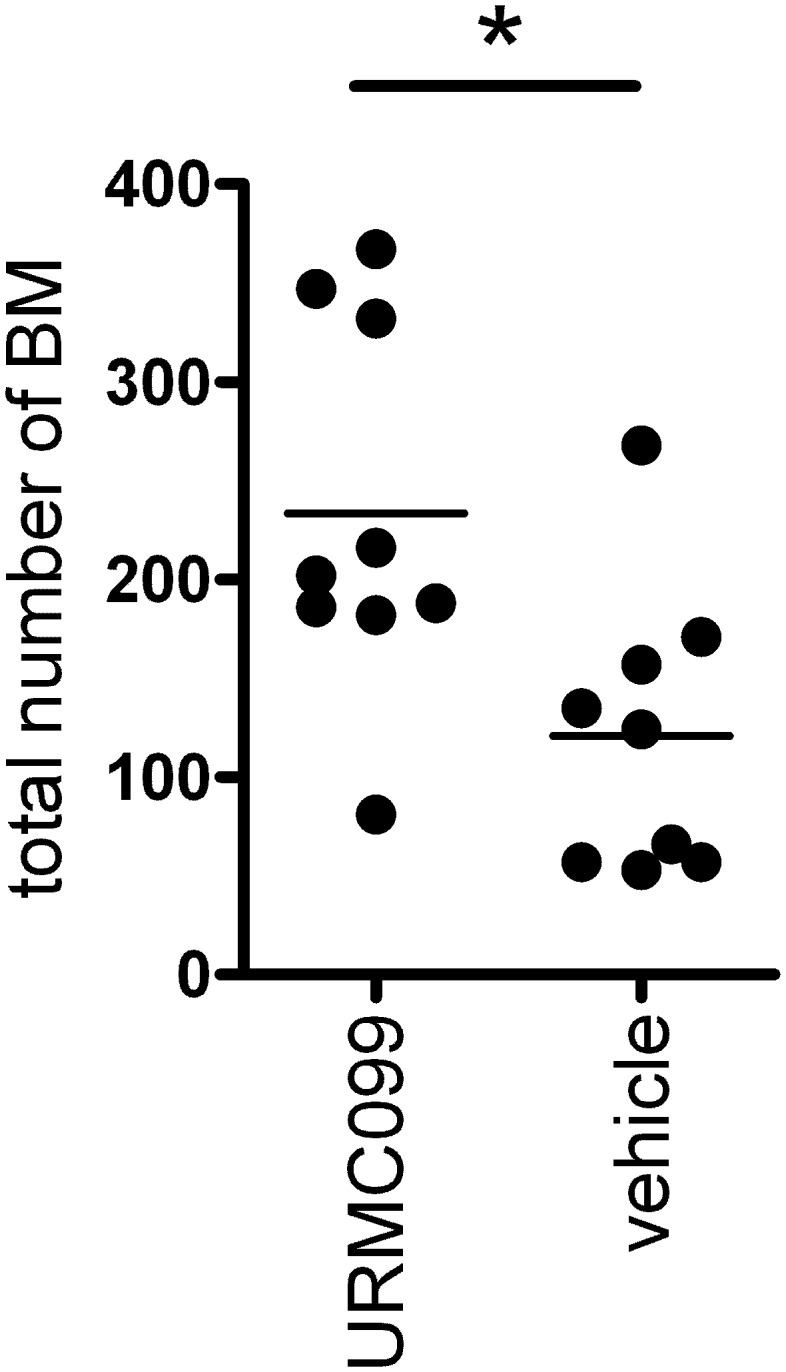
Total number of brain metastases is not reduced in mice treated with URMC099. The total number of metastases was counted in 8 serial coronal sections of each brain. Each dot represents an individual mouse. The bars represent mean values. Asterisk denotes statistically significant difference (p<0.05, two-tailed t-test).

We determined whether treatment with URMC099 altered the number of BM per mouse, by counting the number of BM (number of distinct, eGFP positive cell clusters) in 8 serial sections from each brain. Most of the BM occupied an area smaller than 50 µm^2^, likely representing single cells or a small number of cells [18], [24], and were considered micrometastases. Metastases which had >50 µm^2^ area were considered macrometastases, and represented a small fraction (1–10%) of the total number of metastases. This is consistent with previous reports which use this model [18], [25]. We found that the total number of brain metastases was not reduced in mice treated with URMC099. Unexpectedly, URMC099 treatment significantly (p<0.05, two-tailed t-test) increased the total number of BM in mice (Figure 4). For micrometastases, the pattern is similar to that observed for total BM (Table 1). The number of macrometastases was statistically indistinguishable between mice treated with URMC099 or vehicle (Table 1). We also examined the size of the macrometastases, and found that it was similar for mice treated with URMC099 or vehicle (Table 1).

**Table 1:** Micro- and macro-metastases in mouse brains.

Trearment		Number of micrometastases,per mouse		Number of macrometastases,per mouse		Size of macrometastases,per mouse	
	N	mean	SD	mean	SD	mean	SD
URMC099	9	223.0**	92.79	10.44	3.81	92.73	14.84
vehicle	9	112.4	65.24	7.11	4.34	129.10	107.80

**the number is significantly (p<0.01, two-tailed t-test) different from vehicle-treated group.

Collectively, these data show that URMC099 had no significant effect on reducing either the frequency or size of breast cancer brain metastases in our mouse xenograft model.

## Discussion

MLK3 has been previously identified as a possible therapeutic target for metastatic breast cancer because of its critical role in migration and invasion of breast cancer cells [8], [11], [15]. We therefore hypothesized that inhibition of MLK3 with the novel, brain-penetrant MLK3 inhibitor URMC099 [17], [26] might reduce migration of breast cancer cells *in vitro* and prevent formation of brain metastases *in vivo*.

We found that URMC099 reduced the migration of triple negative breast cancer cells in an *in vitro* scratch wound healing assay and a transwell migration assay, in a dose-dependent manner. These data are consistent with previous results, showing that pharmacological inhibition of MLK3 can reduce *in vitro* migration of breast cancer cells [8], [15].

We also found that URMC099 dose-dependently reduced the migration of normal breast epithelial cells (MCF-10A). We attribute this to the fact that MCF-10A cells also express MLK3, though at lower levels than MDA-MB-231 breast cancer cells [8], and conclude that MLK3 activity may be necessary for normal migratory activity of breast epithelial cells.

Inhibition of MLK3 with URMC099 did not significantly change the growth rate of MDA-MB-231 and its subline, eGFP8.4, which we used in our *in vivo* studies - nor did it cause any obvious increase in cell death. This contrasts with earlier studies, which showed that shRNA-mediated knockdown of MLK3 led to a decrease in the *in vitro* growth rate of MBA-MB-231 cells and an increase in cell apoptosis [11]. One explanation for this difference is that URMC099 inhibits only the kinase activity of MLK3, whereas shRNA-mediated knockdown will affect both kinase activity and the physical scaffolding functions of MLK3 (by depleting the total amount of protein available in the cell). Alternative explanations include the fact that we used a modified, brain-homing subline of MBA-MB-231 cells in our study [18], and that the off-target effects of URMC099 and MLK3 shRNAs are likely quite different [17], [26]. Off-target effects of shRNA-mediated knockdown include potential competition with cellular miRNA/shRNA pathways [27], silencing of untargeted transcripts due to short regions of sequence complementarity [28] and NFκB activation [29]. In contrast, URMC099 – like many other kinase inhibitors - has inhibitory activity against several non-target kinases, including LRRK2 and Flt3 which are also implicated in cell migration [17], [26].

Finally, we examined the effect of MLK3 on breast cancer breast metastasis using a well-defined and widely used mouse xenograft model developed by Steeg and colleagues [18], [25]. In this model, brain-seeking MDA-MB-231-BR human breast cancer cells with affinity for the CNS microenvironment are introduced by direct intracardiac injection into immunodeficient mice, and brain metastases are counted 21 days later. This simplified *in vivo* paradigm models the key, rate-limiting extravasation step of brain metastasis.

We found that URMC099 had no effect either on the incidence of brain metastases (i.e., the percentage of mice that developed brain metastases), or on the size of brain metastases per animal. Interestingly, we observed that treatment with URMC099 was associated with an increased number of micrometastases in mice. The reason for this is unclear at present, and needs further investigation. Thus, the effects of URMC099 in the *in vitro* scratch wound healing assay failed to predict *in vivo* effects on brain metastasis, This may reflect the fact that the complex *in vivo* metastatic cascade cannot be modeled adequately by studying the migration of cells in culture.

Previous studies have shown that shRNA-mediated knockdown of MLK3 can prevent the *in vivo* metastasis of MDA-MB-231 cells from the breast fat pad to the lung [15] and to distant lymph nodes [11]. However, our findings suggest that blockade of MLK3 kinase activity has no effect on *in vivo* metastasis of MBA-MB-231 cells to the brain, following direct intracardiac injection. Importantly, we have previously demonstrated that URMC099, when delivered to mice at the same dose used in our study, penetrates the BBB and reduces the activity of a downstream target of MLK3 (JNK) in brain tissue [16], [17]. Thus, we can assume that URMC099 successfully inhibits MLK3 kinase function in mouse brain in the present experiments. One explanation for the failure of this MLK3 inhibitor to reduce brain metastases *in vivo* is therefore that the physical scaffolding properties of MLK3 - rather than its kinase activity – may be crucial for cell migration *in vivo*.

In summary, we have shown that a novel, brain penetrant MLK3 inhibitor (URMC099) can reduce the migratory activity of breast cancer cells and normal breast epithelial cells in an *in vitro* scratch wound healing assay and an *in vitro* transwell migration assay. However, URMC099 had no effect on *in vitro* cell growth or on the frequency or size of breast cancer brain metastases, when tested in an preclinical mouse xenograft model. These findings suggest that the inhibition of MLK3 kinase activity may not be an ideal stand-alone therapeutic target for the prevention and treatment of breast cancer brain metastasis.
